# The influence of model choice and input data on pollinator habitat suitability in the Hannover region

**DOI:** 10.1371/journal.pone.0305731

**Published:** 2024-09-27

**Authors:** Malte Hinsch, Jens Groß, Benjamin Burkhard

**Affiliations:** Institute of Physical Geography and Landscape Ecology, Leibniz University Hannover, Schneiderberg, Hannover, Germany; Instituto Federal de Educacao Ciencia e Tecnologia Goiano - Campus Urutai, BRAZIL

## Abstract

The habitat suitability for pollinators is an important factor for biodiversity and crop-based ecosystem services. Most flowering plants, including wild plants, rely on pollination ecosystem services for fructification and reproduction. Suitable nesting sites and accessible floral food resources are crucial to the abundance of pollinator insects. Therefore, the suitability for pollinators and the pollination service itself are influenced by the type of land use, the composition of the land cover and structures in the landscape. One way to estimate pollinator habitat suitability is to use computer models such as ESTIMAP and InVEST. Both models calculate the habitat suitability based on spatial land use data and their suitability as nesting and feeding habitats. Besides the decision for a model, the selected spatial dataset also has important effects on the modelling results. In Germany, a large number of Land Use and Land Cover (LULC) datasets is available, such as the European CORINE Land Cover, CORINE Urban Atlas, Biotope types, ATKIS or Open Street Map. These datasets differ in terms of spatial and thematic resolutions, LULC types, and abundance of structural elements, which are crucial input factors for modelling with ESTIMAP and InVEST. We applied and tested both models on the basis of two different datasets in the study area Hannover region in central Germany. A literature-based estimation and expert-based questionnaire determined the biophysical properties required for modelling with ESTIMAP and InVEST. The differences between the results based on two different spatial datasets and the differences between the models were estimated and the results that can be obtained by using freely available data were investigated and compared with the results based on non-publicly available data. The comparison of the results shows that the proportion of near-natural structures in the landscape are a decisive factor for modelling results. The comparison of the models shows that ESTIMAP estimates a higher influence of small structures in the landscape than InVEST, resulting in a higher pollinator habitat suitability. The median similarity index of the two models is between 0.68 to 0.93 for the highly detailed biotope type dataset and 0.40 to 0.79 for the less detailed Corine dataset. The results provide a guidance on how to choose the appropriate model and data to assess pollination ecosystem services.

## 1. Introduction

Pollination regulating Ecosystem Services (ES) are an essential contributor to biodiversity and related provisioning and cultural ES supply. Pollination describes the reproduction of plants based on pollen-transfer by animals, wind and water [1–3]. 87% of flowers worldwide depend on pollination by animal pollinators [1]. This means, pollination corresponds directly to the abundance and diversity of pollinators [4, 5]. In Europe, more than 80% of crops benefit from pollination, an ES with a monetary annual value that has been calculated to be 14 billion Euro in Europe [1, 6]. As a consequence of anthropogenic impacts, such as intensification of agriculture, land use changes or increasing traffic, the abundance of pollinator insects is threatened [3, 7–9]. Pollination of plants by animals and therefore the pollination ES is an elementary service provided by an ecosystem and is thus a core component of the ES concept [4].

The ES concept has become one of the most frequently used frameworks to qualify and quantify pollination as an ES. Within the ES concept, the condition of ecosystems and natural capital are the base of human well-being, which depends on biodiversity and the functionality of ecosystems and thus on the structures and processes of an ecosystem [1, 2, 6, 8, 10, 11]. According to the CICES classification, pollination is listed as a regulating ES [11]. Pollination ES assessment and valuation as a tool can therefore be used to inform stakeholders and develop strategies in various fields, such as nature conservation, research and education, resource management and environmental management, urban and regional planning [10]. To protect pollinator species and therefore biodiversity, special attention should be given to pollinator habitat suitability and thus the potential pollination performance in landscapes [6, 12].

The most accurate method to estimate pollinator habitat suitability still requires pollinator identification directly in the field. However, this task is labor-intensive, largely site-specific and impractical over larger areas. Furthermore, the trapping and killing of insects is an invasive intervention in the ecosystem. Hence, various pollination models have been developed to estimate pollinator habitat suitability and/or pollination performance [6]. Geographic Information Systems (GIS) have proven to facilitate such modelling. ES models such as ESTIMAP (Ecosystem services mapping at European scale) (https://publications.jrc.ec.europa.eu/repository/handle/JRC89594) and InVEST (Integrated Valuation of Ecosystem Services and Tradeoffs) (https://naturalcapitalproject.stanford.edu/software/invest) were developed based on GIS in order to depict indicators for specific ES. For example, the habitat suitability for pollinator species was used as a proxy to indicate pollination ES potential. Most of these models are based on spatially explicit LULC data and ecological or socio-economic data. In addition, the ability to predict and calculate ES with actual measured or evaluated data allows to estimate ES in larger or inaccessible areas. These data are collected, for example, through expert interviews. Furthermore, it is possible to create future scenarios to predict changes in the habitat suitability and therefore the possible abundance of wild bees. [10].

The relatively high number of available ES models and the associated differences in model properties and internal model processes make it difficult to choose a suitable model. In addition, data and resource availability, technical capacities and, last but not least, the actual purpose of the modeling are also important parameters. Several operational approaches have been developed to support the ES model selection process [13–15]. However, not only the choice of the model, but also the choice of suitable geodata for the modelling is crucial. In Germany, various LULC datasets are available, for example the European CORINE Land Cover [16], CORINE Urban Atlas [17] and ATKIS (German Authorative Topographic-Cartographic Information System, Digital Landscape Model at a scale of 1:50000 [18], Biotope type data [19] or Open Street Map (https://download.geofabrik.de/).

All these datasets contain different spatial, temporal and thematic resolutions and include different quantities of landscape structure classes. Linear landscape structures like hedgerows, tree groups or field margins exhibit greater plant diversity, especially in agricultural regions and can serve as nesting sites, food sources and stopovers for animals. They are therefore particularly important for pollinator insects and therefore crucial for the modeling process. Knowledge of these landscape structures is therefore essential for estimating pollinator habitat suitability [4, 20].

A better understanding of these models and their underlying modelling purposes can lead to more accurate ES maps and hence to a more detailed estimation of ES. Robust models are especially needed for pollination ES, because pollinator habitat suitability is of increasing ecological and societal relevance while insect and pollinator biodiversity is declining globally. Human food security depends on pollination, and this knowledge can provide useful for environmental management and landscape planning. [3, 7–9].

The objective of this study is to estimate the influence and dependence on input data for the modelling of pollinator habitat suitability with ESTIMAP and InVEST. Therefore, we methodically assess the performance of these two (at least in Europe) most popular pollination ES models by applying ESTIMAP and InVEST in the same regional case study area with similar model assumptions and input data. The dependence of the models on LULC data and the associated quantity of included structures like hedges and small paths in the landscape was also tested. Both models are comparable regarding their basic assumption that pollination ES can be calculated based on habitat suitability for nesting and foraging of relevant pollinator species. More detailed descriptions of the models, similarities and differences are presented in Section 2.4. The models are applied in the context of potential habitat suitability for wild bees. Another main objective of the study is to help ES modellers to select the appropriate model and input data for their specific ES studies on pollination.

This leads to three questions:

1. What are differences and similarities between the two pollination models ESTIMAP and InVEST?
2. How do different input data affect both models’ performance and outcomes?
3. Which model is suitable for which application and user needs?

## 2. Materials and methods

ESTIMAP and InVEST calculate pollinator habitat suitability based on LULC-specific nesting availabilities (NA), flower availabilities (FA) and an average flight distance of the estimated pollinator species (see Section 2.4 for detailed descriptions of both models). ESTIMAP and InVEST were chosen because both models determine habitat quality and therefore provide comparable results. Furthermore, both models have similar data requirements. In order to assess the functions and differences of ESTIMAP and InVEST, both models were applied in the same study sites with identical input data. Furthermore, the influence of the input data (the Biotop Class Dataset and the CLC Class Dataset; Section 2.2) on both model performances and outcomes was investigated by using two different datasets for the same study site.

### 2.1. Study site

**Fig 1:**
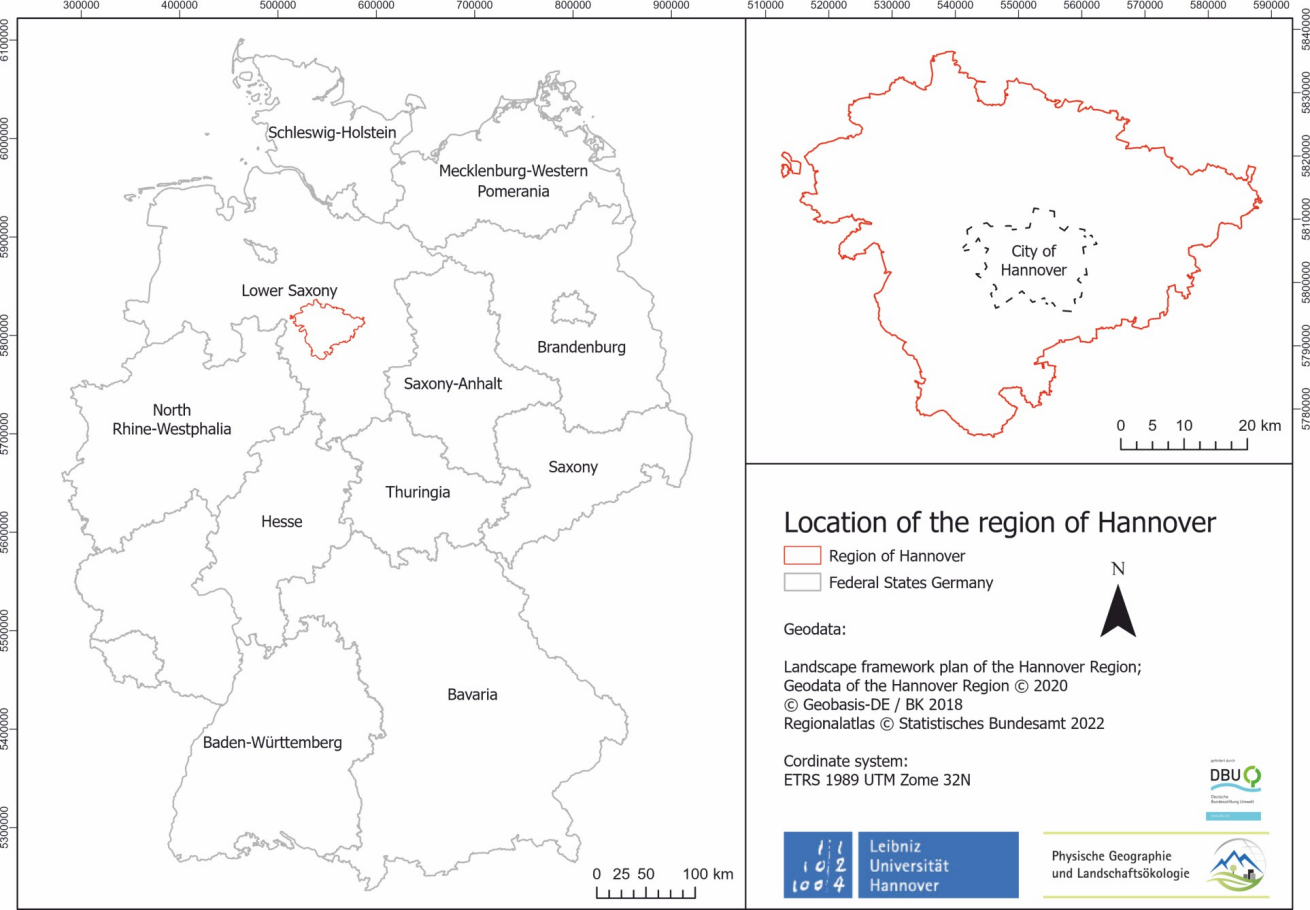
Research area Hannover region [18].

The assessment was conducted in the Hannover region in Lower Saxony in central-northern Germany. The study area comprises 2296,8 km^2^ [19] (Fig 1). Major LULC types include urban areas, agricultural areas, forest ecosystems and waterbodies, for example the river Leine and natural and semi-natural areas such as natural grasslands. Semi-natural areas have a higher potential for the occurrence of wild bees [21], because different wild bee species have different flower preferences and different nesting requirements. Areas with a wide variety of flowers and small structures are more suitable as habitat for wild bees [27]. Due to the high variety of LULC types in this region, the impact of different forms of LULC data can be estimated relatively easily. The major crops in the study region are grass, barley and wheat [22]. These three crops provide pollen for some insects [23], but do not provide nectar. Furthermore, these plants are not primarily dependent on insect pollination. This implies that large parts of agricultural areas are not primarily dependent on pollination, but can still derive a benefit from it [23].

### 2.2. Geodata

**Fig 2:**
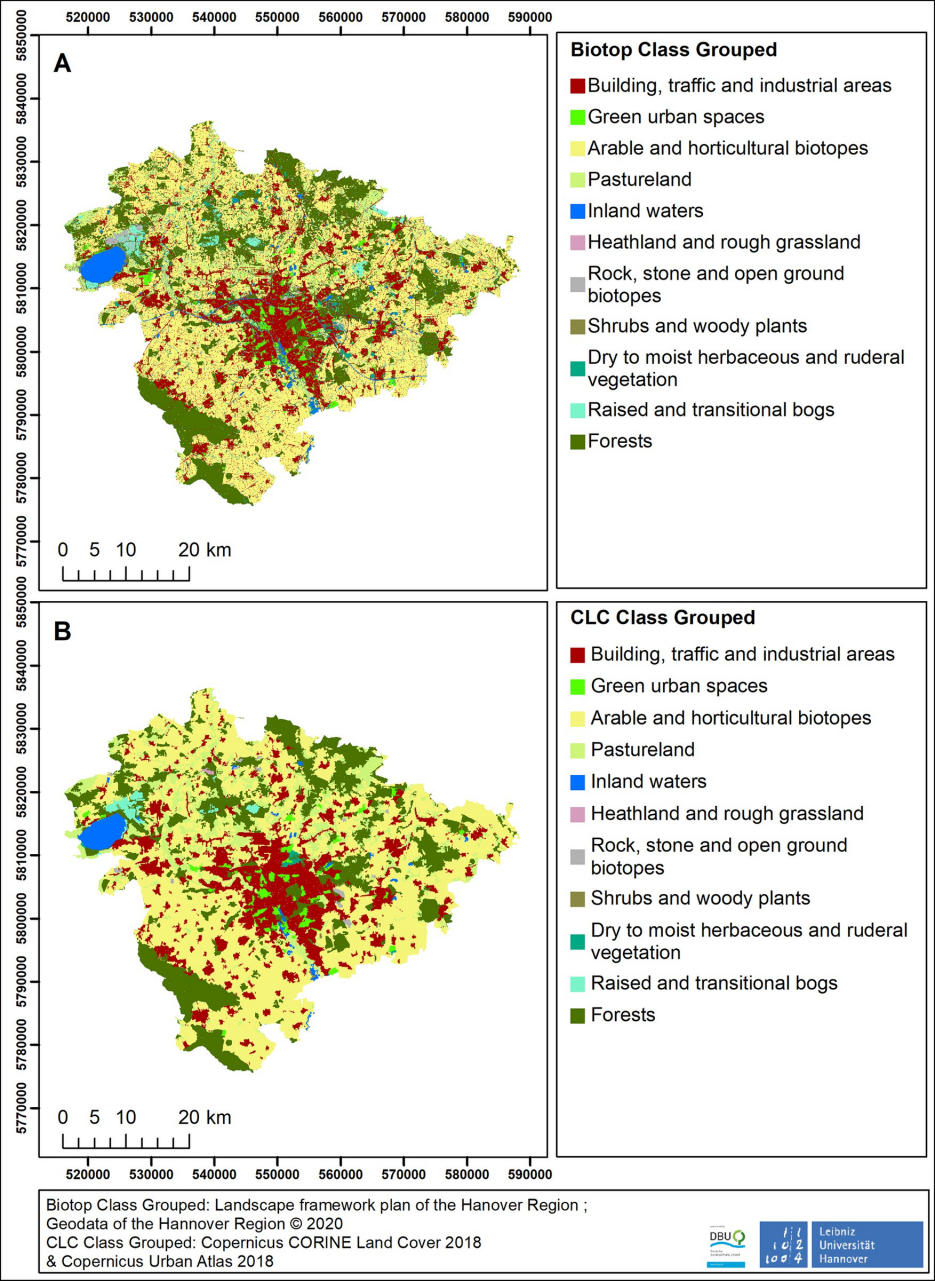
(A) The Biotope Class Dataset consisting of the biotope type groups of the Landscape framework plan of the Hannover region [19]. (B) The CLC Class Dataset 2 consisting of the CORINE Land Cover in rural areas [14] and the Copernicus Urban Atlas in the urban area [17].

The model results were generated and compared on the basis of two different LULC datasets. In Fig 2, a grouped representation (Biotope Type Grouped and CLC Class Grouped) (S2, S3 Tables) of the input data is provided. This grouping is only for a simplified overview and later analysis. The modelling in this work was carried out with the more precisely classified data (Biotope Class (S2 Table) and CLC Class (S3 Table)).

**The Biotop Class Dataset:** This land use dataset is based on the Biotope types of the Landscape framework plan of the Hannover Region, which is not publicly available. Biotope types contain mostly detailed information about the potentially occurring plant communities and other typical features [19]. The biotope data of the Hannover region area under consideration comprise 13 superordinate groups, 128 main units and 554 sub-units as well. The higher-level groups are thus hardly differentiated, whereas the sub-units are very detailed. In order to ensure comparability of the different classes, it is important that the evaluation of the classes takes place on the same level. Since the subclasses are not available in the same detail for all biotope types, it was decided not to use them, even though they show the greatest level of detail for the natural areas. For this reason, the main unit level was used for modeling. However, some main units are combined into classes according to the relevance and frequency of individual areas or biotope types. In total, the modeling was therefore carried out with 84 different units, referred to as biotope classes in the following. S1 Table in the Supplementary information shows the breakdown of the biotope types and the resulting 84 selected classes. Since forest edges are an integral part of the ESTIMAP modelling process, each forest area was adjusted with a 15 meter forest edge. Such a forest edge was created at each contact zone between a forest type and another biotope class (including other forest types). Riparian zones with a buffer of four meters were created manually on the borders to rivers and lakes. This pre-processing was done with Microsoft EXCEL and ArcGIS, more precisely with the "Cut", "Merge" and "Delete" tools of ArcGIS to create a detailed dataset that best reflects the heterogeneity of the study area. For reasons of clarity, the biotope types were divided into 11 groups. S2 Table in the Supplementary information shows the allocation of these groups and the corresponding biotope types. This dataset is referred to as the Biotope Class Dataset **(**Fig 2A).

**The CLC Class Dataset:** The second LULC dataset is based on a combination of the CORINE Land Cover 2012 data (CLC) [16] and the Copernicus Urban Atlas 2012 (UA) data [17].

CLC is available for 48 countries and covers the entire continental Europe. It contains altogether 44 LULC classes (https://land.copernicus.eu/user-corner/technical-library/corine-land-cover-nomenclature-guidelines/docs/pdf/CLC2018_Nomenclature_illustrated_guide_20190510.pdf), such as “complex cultivation patterns”, “land principally occupied by agriculture” to “non-irrigated arable land” [16]. The UA data has more specific land use classes (https://land.copernicus.eu/user-corner/technical-library/urban-atlas-mapping-guide) within urban areas, such as “continuous urban fabric”, “discontinuous dense urban fabric” and “discontinuous medium density urban fabric” [17]. For this reason, a dataset was created in which the rural areas in the CLC dataset were merged with the urban areas in the UA dataset. UA was used for all urban areas that were available for the study area. All other areas were filled with the CLC data. The intention was to create a detailed dataset, based on open access available data, that reflects the heterogeneity of the study area. Because both, CLC and UA data, do not provide data on forest edges, forest edges of 15 meters were computed manually using the ArcGIS *buffe*r tool. Alongside the forest edges, riparian zones with a buffer of 5 meters were added. After the selection of the forest edges and riparian zones, the appropriate LULC classes were clipped and then merged with each other. Fig 2B presents the CLC Class Dataset.

### 2.3. Biophysical data

**Reference species:** To represent the different nesting and food requirements of the various wild bee species, the modelling was carried out using an “reference species” as an example. The reference species represents the average characteristics of 44 selected wild bee species with their mean flight distances. Honey bees are not considered further for this study, as their nesting suitability depends more on human influence and the provision of hives than on ecosystem conditions. Furthermore, bumblebees were not considered further, as their potential flight range is far greater than that of other wild bee species, and thus a common model is not possible. For the InVEST modelling, we used a parameterization of wild bee characteristics that includes information on nesting suitability, foraging activity, flight range and relative abundance. Here we assumed that the reference species can nest in both soil and structures. Therefore, for the InVEST modelling, the highest possible value of 1 was assumed as the optimal value for both nest cavity availability (nesting in structures such as plant stems and walls) and nest site availability (nesting in the ground). Furthermore, it was assumed that the reference species covers both spring and summer, as the different underlying wild bees can be found in the different periods. A mean flight distance of 680 m was determined for the reference species. The wild bee species considered for the modelling are listed in the supplemental information (S4 Table).

Both models (ESTIMAP and InVEST) calculate the potential habitat suitability for pollinators partly based on the presence of nesting sites and food resources for specific pollinators [21, 24]. For each dataset, the information on existing nesting sites and food availability was obtained differently.

The assessment of biophysical land cover attributes for the Biotop Class Dataset was based on a semi-quantitative expert questionnaire. The purpose of the questionnaire was to estimate the local availability of flowers (FA) and the suitability of breeding areas (NA) for each LULC class within the study region. The biophysical assessment was carried out in collaboration with a total of 10 wild bee experts using a questionnaire developed for this study. In addition to the questionnaire, a definition of the individual biotope types with information on the indicator plants (if available) and a map of the biotope types in the Hannover region were provided to assist the assessment. The aim was to evaluate each LULC individually in terms of nesting suitability and food availability. The expert-based rating was given from 0 = (no availability / suitability) to 10 = (highest availability / suitability). Resulting ratings were transformed to an index ranging from 0 to 1, which is required by ESTIMAP and InVEST [21, 24]. S5 Table gives an overview of the expert survey with all biophysical data according to the Biotope Class Dataset.

The biophysical land cover attributes for the CLC Class Dataset were based on the ESTIMAP documentation: "ESTIMAP: Ecosystem Services Mapping at European level" [21]. In the documentation, standard values for NA and FA are proposed. These data were assigned to the land use classes of the Copernicus Dataset. The FA and NA data of the ESTIMAP documentation [21] are initially based on CLC Data classes and respective FA and NA assessments at European level, hence they are not specific for the Hannover region. Due to the use of UA Data as part of the CLC Class Dataset, some classes do not directly conform to the ESTIMAP documentation. An interpolation was needed to create NA and FA values for each of the classes that do not accord to the CLC Class Dataset. Discontinuous urban fabric is defined as having a building density of more than 30%, continuous urban fabric corresponds to a building density of more than 80% respectively [25]. Furthermore, the discontinuous dense urban fabric, with a density of 50% - 80%, received FA and NA between these classes. The same applies to discontinuously dense urban fabric, with a density of 10% - 30% and discontinuous dense urban fabric with a density of less than 10% [25]. For these classes, values were assigned that lay in-between the values of the original discontinuous urban structure data and the next logical class from the original CLC dataset. Because of the lack of transferable data for the UA classes "express roads and associated land", "other roads and associated land" and "railways and associated land", these classes were merged into one LULC class. S6 Table lists all biophysical data based on [18] according to the CLC Class Dataset.

### 2.4. Pollination models

**ESTIMAP** is a modelling framework to assess ES at the European scale. It contains different modules, such as an outdoor recreation module, a coastal protection module, an air quality regulation module and a pollinator habitat suitability module [6, 26]. The ESTIMAP pollination model was developed in 2013 by the JRC (Joint Research Center) of the European Commission to assess the potential contribution of pollinators to agricultural production across Europe. The module is based on expert assessments of land use classes in terms of nesting site availability and food supply to calculate pollinator habitat suitability. This habitat suitability is provided in the form of a dimensionless index [21]. In addition, the potential flight distance of pollinator insects using a distance function is used to evaluate the distance of potential nesting and foraging habitats. [3]. Here, the distance function represents a positive effect on habitat suitability for pollinators from semi-natural areas and forest edges in the study region. Necessary data for modelling with ESTIMAP are two grids that contain the values for nesting suitability (NA) and the floral availability (FA) as a floating number between 0 and 1 (0 = no nesting sites or floral resources and 1 = the best possible nesting sites/floral resources in the study area). The model requires geodata-processing software such as ArcGIS or QGIS. The ESTIMAP model procedure leads to two different results: 1) The potential suitability of the landscape for the presence of pollinator insects; and 2) The dependence of the investigated pollinators on certain flowering plants [21]. We conducted the ESTIMAP modelling process with a mean pollinator flight range of 680 m. The flight distance of the models is based on the flight distance of a reference species. Since ESTIMAP was first developed for continental evaluation [21, 27], an activity index was introduced to consider the radiation and temperature differences in larger areas [21]. At the local or regional level, such differences are usually negligible. Hence, an addition of the activity index was not needed in this study.

**InVEST** is an open source and GIS-based modelling framework developed in 2008, containing more than 20 ES modules [24]. The framework estimates different ES or ES indicators to quantify and map ES based on spatial and/or statistical data. The InVEST pollinator habitat suitability module calculates the pollination supply and the pollinator habitat suitability based on three main factors, similar to ESTIMAP [24]. Although the model focuses on wild pollinator species, the pollinator potential of managed bees for crop yield can be manually adjusted [24, 28]. Model input requirements are: 1) LULC data in grid format; 2) A CSV table with biophysical land cover attributes, containing the land use code and the availability of nesting habitats (cavity and ground). These values range from 0 (no suitability) to 1 (full suitability). In addition, the CSV table includes values to floral resources for the different seasons, also on a scale of 0 = no availability to 1 = highest availability) [20, 24]. 3) Species information as a CSV table [20]. In this study, a reference species species is used as a pollinator for modelling with InVEST, containing the average characteristics from 44 different wild bee species. A list with the bee species is given in S4 Table.

Both models are similar in their aims and input parameters, but the modelling workflows are different. The main difference between the two models is the underlying calculation design. ESTIMAP exploits the presence of semi-natural surfaces as a positive influence on other areas. This is represented by applying an exponential buffer starting from certain favorable areas and extensive potential flight distance. InVEST evaluates an area in relation to the initial assessment of the surrounding areas. This is applied by offsetting the valuation of surrounding areas against the area under investigation. Another (technical) difference between the two models is that a GIS software is needed to actually run ESTIMAP, whereas for InVEST, a GIS software is only needed for data preparation. In this respect, the implementation of ESTIMAP requires a higher level of knowledge about the handling of geospatial data in geographic information systems than InVEST.

### 2.5. Map comparison

A similarity index was calculated in order to compare the outcomes of the two models’ and the two datasets. This index is based on the theory of the cell-by-cell numerical comparison method of the Map Comparison Kit [29] and shows the degree of deviation of a grid cell of each map. The similarity index was calculated with the ArcGIS Map 10.6 Raster Calculator. An additional step was performed to change the absolute difference into an absolute similarity. Here, 1 means absolutely identical and 0 means absolutely different. The formula (Formula 1) below shows the calculation of similarity. The absolute difference between the two different maps (a and b) is calculated. Furthermore, the difference is converted into a similarity by calculating 1—the difference.

**Formula 1.** Similarity (S) of both maps based on the Map Comparison Kit [29].


S=1–con(a−b<0,b−a,a−b)

For the reason of clarity, the LULC classes were divided into groups. A table with the exact order for each LULC class can be found in the Supplementary information.

## 3. Results

### 3.1. General model results

Table 1 shows the average habitat suitability for each model / dataset combination of the entire study area. The ESTIMAP model does not show a large deviation between the mean habitat suitability of the two data sets. However, a deviation can be seen in the standard deviation. This deviation is larger for the CLC Class dataset. With the InVEST model this is exactly reversed. Here the Biotop Class dataset has a 10% higher habitat suitability than the CLC Class dataset. However, the standard deviation is hardly different.

**Table 1:** Habitat suitability (median) and standard deviation for each model and each dataset for the entire study area of the Hannover Region.

Model	Dataset	Habitat Suitability (median)	Standard devations
ESTIMAP	Biotop Class	0.40	0.11
	CLC Class	0.39	0.21
InVEST	Biotop Class	0.22	0.13
	CLC Class	0.12	0.14

Tables 2 show the median habitat suitability and the median similarity between the modelling results values of each LULC class group. The modelled pollinator habitat suitability shows that Rock and stone biotopes (Table 2) have the lowest modelled habitat suitability. In the case of bog biotopes, the first major difference between the datasets becomes apparent. A higher value was calculated for the CLC Class Dataset (Table 2). Areas with a greater human impact, such as agricultural land, buildings, roads and urban green areas, are again in a similar range. The highest habitat suitability was achieved in more natural and semi-natural areas such as forests, shrubs and woody plants and herbaceous and ruderal vegetation. The highest habitat suitability in both datasets was found in heathlands and rough grasslands (Table 2).

**Table 2:** Thematically grouped ESTIMAP and InVEST model results and similarity between model results for the biotop class dataset and the CLC class. The ESTIMAP and InVEST model results are presented as a dimensionless index between 0 and 1, as is the similarity index. The results were generated for the region of Hanover.

LULC Class Group	ESTIMAP habitat suitability (median) Biotop Class Dataset	InVEST habitat suitability (median) Biotop Class Dataset	Similarity Index Model (median) Biotop Class Dataset	ESTIMAP habitat suitability (median) CLC Class Dataset	InVEST habitat suitability (median) CLC Class Dataset	Similarity Index Model (median) CLC Class Dataset
Building, traffic and industrial areas	0.35	0.03	0.76	0.27	0.06	0.79
Green urban areas	0.49	0.27	0.75	0.32	0.01	0.71
Arable and horticultural biotopes	0.35	0.18	0.84	0.30	0.06	0.77
Pastureland	0.37	0.20	0.83	0.36	0.07	0.72
Heathland and rough grassland	0.81	0.61	0.76	1.00	0.60	0.60
Rock, stone and open ground biotopes	0.17	0.04	0.87	0.28	0.02	0.64
Shrubs and woody plants	0.50	0.32	0.83	0.97	0.38	0.40
Dry to moist herbaceous and ruderal vegetation	0.81	0.50	0.68	0.95	0.47	0.52
Raised and transitional bogs	0.24	0.19	0.93	0.59	0.24	0.64
Forests	0.46	0.36	0.89	0.70	0.27	0.61
Inland waters*	0.00	0.00	1.00	0.00	0.00	1.00

*Inland Water was not considered further in the following analyses, as it has no relevance for the habitat suitability of wild bees

### 3.2. Model comparison

**Fig 3:**
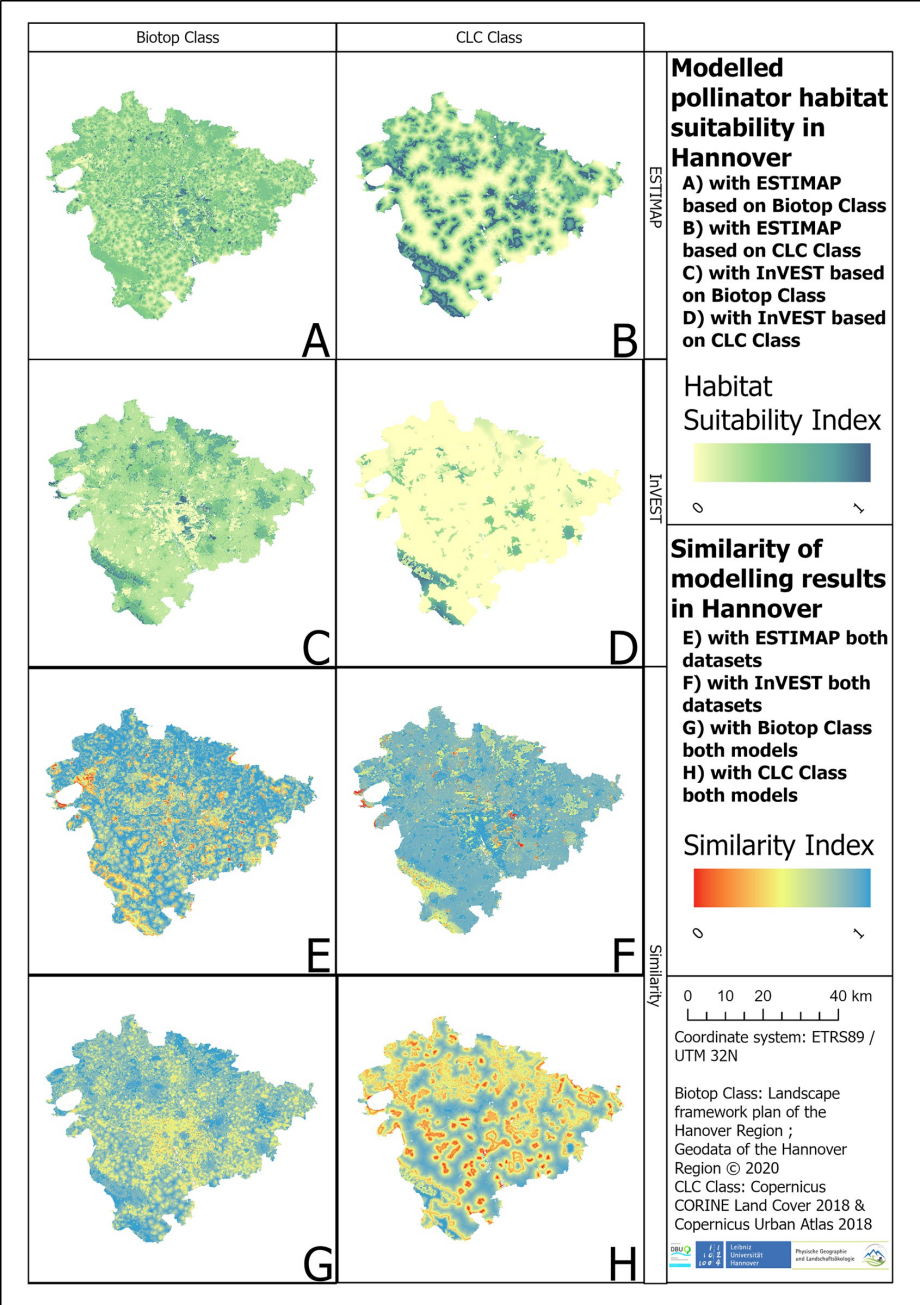
(A) Pollinator habitat suitability modelled with ESTIMAP based on the Biotope Class Dataset; (B) Pollinator habitat suitability modelled with InVEST based on the CLC Class Dataset; (C) Pollinator habitat suitability modelled with InVEST based on the Biotop Class Dataset; (D) Pollinator habitat suitability modelled with InVEST based on the CLC Class Dataset; (E) Similarity between both Dataset results modelled with ESTIMAP; (F) Similarity between both Dataset results modelled with InVEST; (G) Similarity between both model results based on the Biotope Class Dataset; (H) Similarity between both model results based on the CLC Class Dataset; for the Region of Hannover.

**Biotope Class:** This model comparison based on Biotope Class (Fig 3) showed that the ESTIMAP model run had a median habitat suitability value in the whole study area of 0.39 and a standard deviation of 0.12 (in Fig 3A). The InVEST model showed an median pollinator habitat suitability value of 0.22 and a standard deviation of 0.13 (in Fig 3C). The median similarity of both models (Fig 3G) is 0.82, indicating a 18% difference with a standard deviation of 0.10. The ESTIMAP assessment (Fig 3A) showed the highest pollinator habitat suitability in heathland and rough grassland areas towards the east of the city of Hannover, and in forest areas in the southern part of the study area. The lowest pollinator habitat suitability was modelled in the inner-city center of Hannover, especially on inner city streets.

In comparison, the results based on InVEST (Fig 3C) indicated that the estimation of the forest values in the south of the study areas was higher. The lowest values were found in urban areas. However, low pollinator habitat suitability was also found in urban areas, especially in inner city centers.

The calculation of the similarity index between both models based on the Biotope Class Dataset (Fig 3G) showed that the highest similarity was achieved at raised and transitional bogs and forests. These classes had a similarity index ranging from 0.87 to 1.00. The lowest similarity was found in dry to moist herbaceous and ruderal vegetation.

**Fig 4:**
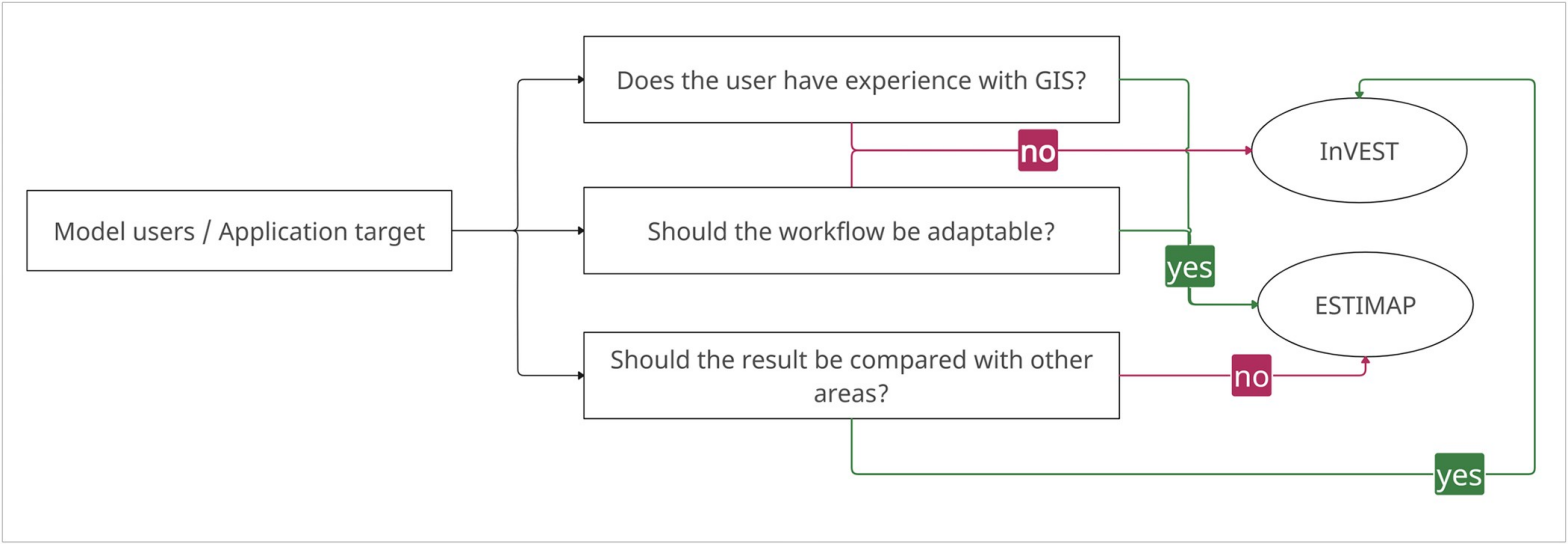
Decision tree for the use of ESTIMAP and InVEST for modelling habitat suitability for pollinators.

**CLC Class:** This model comparison (Fig 3) showed that the mean habitat suitability calculated with ESTIMAP (Fig 3B) was 0.35 with a standard deviation of 0.21. The estimated habitat suitability of InVEST (Fig 4D) had an average pollinator habitat suitability value of 0.12 and a standard deviation of 0.14.

The highest values of the ESTIMAP modelling results (Fig 3B) were found in heathlands and rough grassland, shrubs and woody plants and dry to moist herbaceous and ruderal vegetation. These classes had estimated values of 0.95 and 1.00. Other highly valued land use classes were forest edges in the south of the study area. The forests had a median pollinator habitat suitability of 0.70. The lowest pollinator habitat suitability values were found at streets of the city center.

In comparison with ESTIMAP, the land use class with the highest pollinator habitat suitability value determined by InVEST (Fig 3D) was heathland and rough grassland with an average value of 0.60. The lowest average values referred to water bodies, landfills and areas used by humans such as buildings, roads and urban green spaces (0.00 to 0.06). The values of agricultural areas ranged from 0.06 to 0.07. Therefore, most of the area had a value below 0.10.

The similarity map (Fig 3H) showed the highest values in urban areas (0.71–0.79) and agricultural areas (0.72–0.77). The forest areas and semi-natural areas had a similarity ranging from 0.40 to 0.64.

### 3.3. Dataset comparison

**ESTIMAP:** The dataset comparison done with the ESTIMAP results (Fig 3) showed that Biotop Class Dataset (Fig 3A) had a median pollinator habitat suitability value of 0.39 and a standard deviation of 0.12. The CLC Class Dataset (Fig 3B) had a median pollinator habitat suitability of 0.35 with a standard deviation of 0.21. The similarity between both datasets (Fig 3E) had a median value of 0.83 and a standard deviation of 0.14. Therefore, the ESTIMAP results based on both datasets are identical by 83%.

The highest values of the ESTIMAP results based on the Biotope Class Dataset (Fig 3A) were located in heathland and rough grassland areas towards the east of Hannover, and in forest areas in the southern part of the study area. This assignment is based on the description of the individual LULC classes. The lowest pollinator habitat suitability was found in the inner-city core of Hannover.

In comparison, the highest pollinator habitat suitability values based on the CLC Class Dataset (Fig 3B) were also in the southwest of Hannover, as well as in heathland and rough grassland areas towards the east of Hannover.

The similarity map (Fig 3E) displayed the highest similarity for semi-natural areas like heathland and rough grassland and shrubs and woody plants. The pollinator habitat suitability based on the Biotope Class Dataset located in semi-natural areas (like heathland and rough grassland and dry to moist herbaceous and ruderal vegetation) had a value of 0.81 and the pollinator habitat suitability in semi-natural areas based on the CLC Class Dataset ranges from 0.95 to 1.00. The pollinator habitat suitability values of forests were 0.46 in the Biotope Class Dataset and 0.70 in the CLC Class Dataset. A lower similarity was reached in the forest areas, for example in the south of the study area (Fig 3E), or in the northern parts of the study area, directly in the north of the lake “Steinhuder Meer”, which is mainly covered by raised and transitional bogs (Fig 2).

**InVEST:** The median habitat suitability, calculated with InVEST (Fig 3), based on the Biotope Class Dataset (Fig 3C) had a value of 0.22 with standard deviation of 0.13. Based on the CLC Class Dataset (Fig 3D), the pollinator habitat suitability value was 0.12 with a standard deviation of 0.14. The similarity index (Fig 3F) had an average of 0.86 and a standard deviation of 0.10. Accordingly, both maps are identical by 86%.

In the Biotope Class Dataset estimation (Fig 3C), the highest estimated pollinator habitat suitability values were found in areas in the northeast of Hannover. These areas were categorized as semi-natural areas with an average value of 0.61. Based on the Biotope Class Dataset (Fig 3C), the next highest rated land use classes were heathland and rough grassland (Fig 3C). These classes were located mainly in the southwest (Fig 3C).

The results based on the CLC Class Dataset (Fig 3D) showed similarity to the location of the highest pollinator habitat suitability values. These areas were also located in the northeast of Hannover and in the southwest of the study area. The next highest evaluated area was again dry to moist herbaceous and ruderal vegetation. These areas were located mainly in the south of the study area. The areas with the lowest values were again urban areas like buildings, streets, parks (0.01–0.06) and agricultural areas (0.06–0.07).

The similarity map (Fig 3F) shows that the biggest differences between the two datasets are located outside the city core of Hannover urban green spaces. Another low similarity can be seen in agricultural areas. The semi-natural provided the highest similarity. This high similarity was also achieved by forests and by buildings.

## 4. Discussion

### 4.1. General model results

**ESTIMAP:** The modelling of the possible pollinator habitat suitability of wild bees with ESTIMAP shows that the majority of the study area is moderately to moderately-well suitable as a habitat for the “reference species”. Only the city center of Hannover and water-related areas have no or very low habitat suitability for wild bees. For all other urban LULC types, the habitat suitability is defined by urban sealing, as it impacts the availability of flowering plants and nesting possibilities. The amount of structures in the landscape is also important. Well-structured agricultural areas, for example with forests and extensive green areas, showed a much higher modelled potential than land use classes without such structures. The suitability of forest edges [4, 30] for pollinators is also well-represented in this study, because forest edges have a high potential, though it decreases towards the forest cores, as forest edges have a higher potential for floral resources and open spaces than forest cores [31]. In contrast, the uniform influence of the distance function of ESTIMAP in all land use classes can lead to an overestimation. Due to its adaptability and flexibility, ESTIMAP is particularly suitable for testing different scientific hypotheses and theories.

**InVEST:** The InVEST results on the habitat suitability were similar, especially for natural and semi-natural areas and forests. Although the assessment of urban areas is generally [4] realistic, small-scale structures show little influence on the model results. This is in contrast to the findings of entomologists such as [31] or [4]. In summary, InVEST is well-suited to model large areas with no to very few landscape structures. However, if the influence of these structures is disregarded, the habitat potential can be underestimated. Due to the consistency of the execution of the InVEST model, this model is particularly suitable for the investigation of application-related problems and area comparisons.

### 4.2. Model comparison

**Biotop Class**: The main reason for the different results in The Biotop Class Dataset are the positive effects of semi-natural areas and forest edges that are applied in ESTIMAP. The ESTIMAP modelling reduces the values from the forest areas to the interior of the agricultural fields. The InVEST modelling shows a stronger demarcation between forest and agricultural land uses due to the limited impact of small structures on the habitat suitability. The main estimation is therefore based on the land use class-dependent biophysical attributes [24]. In contrast, every small structure in ESTIMAP shows positive habitat quality effects [21]. In addition, the semi-natural areas and forest edges themselves are rated higher in the ESTIMAP modelling [4], since they are influenced by the distance function as a starting point. This is set-off by the negative influence of roads [32–34], which reduces the adjacent potential presence of pollinators. Hence, the assessment in ESTIMAP depends on the distance to specific land use classes and is less based on the initial assessment of each individual land use class. Therefore, the model presents a more differentiated assessment of the landscape compared to InVEST.

**CLC Class**: The ESTIMAP results show how small forest areas affect the surrounding agricultural area. This is confirmed by the fact that especially forest edges have a high potential as a habitat for pollinating insects [4, 21] and how the value decreases with the distance from these forest edges. InVEST does not detect these removal effects. In this case, the differences between the highly valued forest ecosystems and agricultural land are depicted as hard borders in the maps rather than with a smooth transition. This is due to the higher influence of the initial valuation in the InVEST modelling.

Table 3 compares the application properties and advantages and disadvantages of the two models. Plus (+) here means high properties or requirements and minus (-) low. Plus/minus (+-) shows normal properties or requirements.

**Table 3:** Model comparison for the ESTIMAP & InVEST application.

Model	Possibility to adapt the model code	User-friendliness	Consistence	GIS knowledge required
ESTIMAP	+	-	+-	++
InVEST	-	+	+	-

### 4.3. Dataset comparison

**ESTIMAP:** The dataset comparison for ESTIMAP shows that the greatest differences was reached in the forest areas for example in the south of the study area. As well as in the northern parts of the study area, directly in the north of the lake “Steinhuder Meer”, which is mainly covered by raised and transitional bogs. This can be explained by the fact that the main influence of the ESTIMAP model, apart from the assessment of the biophysical properties, is given by the positive influence of semi-natural areas [6, 26]. The Biotop Class Dataset provides a higher amount of small structures, such as hedges and small forest patches, which have a significant influence on other areas [4, 26]. The amount of these small semi-natural structures is lower in the CLC Class Dataset and therefore, the estimated habitat suitability was lower with the CLC Class Dataset in comparison to the Biotop Class Dataset. The negative influence of roads on the habitat suitability for pollinators is clearly visible with both datasets. This shows that the CLC Class Dataset in urban areas offers a similar number of high traffic roads than the Biotop Class Dataset. This leads to a lower habitat quality due to the limiting effect of roads on pollinator insects [32–34].

**InVEST:** The dataset comparison shows that the outskirts of the city had the highest deviation. The Biotop Class Dataset provides a greater number of urban areas with a lower density in the outskirts of the city than the CLC Class Dataset. These land uses classes are highly suitable habitats for wild bees [4]. Due to the fact that the results of InVEST mainly depend on the biophysical attributes [24], the Biotop Class Dataset is evaluated higher than than the CLC Class Dataset, because the Biotop Class Dataset shows a higher number of classes with a better initial evaluation. For the same reason, there are no significant differences in the rest of the research area, as the presence of local structures (which, as mentioned above, are more common in the Biotop Class Dataset than in the CLC Class Dataset) does not have a significant impact on InVEST modeling. In addition, the negative influence of roads on pollinator habitat suitability [32–34] is also considered with InVEST.

### 4.4. Discussion of the uncertainities

The use of models involves general uncertainties. The first and most crucial point is that a model is always an abstraction of reality and thus can never take all factors into account. Furthermore, a model is based on input data and the parameterization of these data [35].

The positive influence of each semi-natural area in ESTIMAP could lead to an overestimation of the pollination ES potential, since a positive effect is modeled from every edge of the forest, regardless of its actual conditions. Conversely in InVEST, the disappearance of such a positive influence leads to an underestimation. This is shown by the fact that ESTIMAP has a gradient between different LULC types, whereas InVEST implements hard boundaries. It would be important to know whether such hard boundaries are realistic due to a sudden lack of, e.g., food resources or if it is rather a slow decrease of resources and thus a softer transition. Drawing upon highly generalized LULC data instead of vegetation data or at least habitat-specific data for the modelling bears uncertainties. However, due to the lack of other data, it is often the only option, especially when modelling larger areas. The inclusion of more detailed information would improve the assessment of habitat suitability in general [4].

The highest uncertainty of the dataset comparison is that the applied biophysical land cover attributes are not based on the same source for both records. The biophysical attributes for the Biotope Class Dataset were compiled using an expert interview and the biophysical attributes for the CLC Class Dataset are based on literature [21]. This is because an assessment of habitat suitability by local wild bee experts was not possible due to the paucity of information on plant communities and nesting sites based on CLC and UA data. This means that the absolute habitat suitability values can only be compared to a limited extent. However, the influence of the degree of detail by e.g. local structures can also be recognized independently of this factor. A comparison of the two datasets may therefore be based on the presence of specific structures rather than the actual assessment of the areas. The influence of small local structures like hedges, semi-natural areas, woods [4, 26] and roads [32–34] is important to properly estimate the habitat suitability. Therefore, when possible, the most detailed dataset should be used to estimate the pollinator habitat suitability, depending on the modelling scale.

One uncertainty in modelling with both ESTIMAP and InVEST is the use of the predefined LULC types, which do not contain area-wide and detailed information on vegetation and plant types and densities for all classes. However, this information would be useful for estimating an adequate supply of pollen and nectar from certain plants, which again are a precondition for estimating pollinator habitat suitability. As a result, the use of LULC data may lead to even more simplification, which is already caused by the modeling process. For a more accurate estimation, knowledge on the different vegetation types within each land use class would be helpful [4, 31]. Due to the different initial assessment of both data sets with regard to their suitability as nesting and foraging habitats, similar classes may deviate. This also partly influences the modeling results and must be taken into account. The manual implementation of ESTIMAP carries a certain potential for errors and entails a risk of low reliability if conducted by different users, which can cause inconsistencies in the implementation and also in the results. This and the fact that the results of ESTIMAP modelling are relative, makes comparison with geographically diverse areas almost impossible.

An additional uncertainty is the use of a "reference species" for modeling. Although this represents a broader spectrum for habitat suitability, it neglects special requirements that the various individual species may have. This means that different bee species sometimes have very different requirements in terms of nesting opportunities and food supply. Furthermore, the flight distance can differ considerably, which is a decisive factor for the modelling.

Another general uncertainty associated with modeling pollinator habitat suitability with ESTIMAP or InVEST is that the modelling results can only offer the habitat suitability and must not exactly represent reality, as the interaction of bees and their environment is more complex than represented with the few modelling parameters [24]. As a final uncertainty, this study did not exactly validate the mapping data. The model results were checked for plausibility by a literature investigation and the focus of the study is on the model comparison and the influence of the input data.

## 5. Conclusions

Both models follow a similar modelling approach, but differ in their implementation. The rather manual execution of ESTIMAP in GIS allows a transparent implementation and adaptation of the modeling, whereas such a modular adaptation is not possible by the black box system of InVEST. Therefore, the main technical difference between ESTIMAP and InVEST is that in ESTIMAP, every step in the modelling can be changed and adapted. The modular structure of ESTIMAP offers many possibilities to adapt the modelling process to possible local conditions or changes (for example the influence of roads). This manual implementation is based on advanced knowledge of how to use GIS software and, at most, knowledge of programming languages to automate the algorithms. Fig 4 shows a simplified decision tree for the choice between ESTIMAP and InVEST. A user should consider whether one has the necessary GIS skills and whether the modelling needs fundamental adaptations. For example, the choice of favorable areas or limitations can be selected by the user. Furthermore, it must be decided whether a comparison of several locally separated areas is desired and whether the model results should be completely independent of the user’s model idea.

Both models provide plausible results on the habitat suitability. ESTIMAP generally rated habitat suitability higher than InVEST in this study. The suitability of forests was also well-represented, as forest edges have a high potential, though it decreases towards the forest cores. In contrast, the uniform influence of the distance function of ESTIMAP, in all land use classes, can lead to an overestimation. However, since the ESTIMAP results are relative, it is more difficult to transfer the outcomes to other areas.

In summary, InVEST is well-suited for modelling larger areas with comparably less landscape structural elements. Disregarding the influence of these structures, however, can lead to an underestimation of the habitat suitability. InVEST showed such a lower incidence of pollinators in the study area. Only the forest areas and semi-natural areas showed a higher potential. The application of ESTIMAP is best-suited for research purposes. Through according adjustments, the model can be further developed and changed to match specific research needs, data availability and the purpose of the modelling study. InVEST can be recommended for application-oriented tasks, e.g. by governmental or planning institutions, since the results are more consistent due to the fixed model structure. Furthermore, InVEST is more user-friendly, as less GIS knowledge is required for implementation and a training dataset is included for each module, which can be used as a template for building the data. Furthermore, the number of ES that can be modelled is much higher than in ESTIMAP.

The comparison of two different datasets revealed that the number of small local structures within the landscape and urban areas significantly influences the modelling results. The structure as well as the composition and thus the diversity of a landscape have a significant influence on the modelling of pollinator habitat suitability. The modelled pollinator habitat suitability in richly-structured landscapes proved to be higher than in homogeneous landscapes.

The influence of small local structures like hedges, semi-natural areas, woods [4, 26] and roads [32–34] is important to properly estimate habitat suitability. Therefore, when possible, the spatially and thematically most detailed dataset should be used to estimate this suitability, depending on the modelling scale.

The selection of the input land use dataset bears implications for the modelling results–especially the influence of small-scale landscape structures that are important for a realistic estimation of the pollinator habitat suitability [4, 31].

For future work, we recommend to improve the precision of the model results for bee-specific pollinator occurrence. For this purpose, so called eco-profiles could be a suitable tool for various wild bees in order to investigate specific pollinator species habitat suitability. Eco-profiles are developed for species communities with similar requirements for nesting and feeding opportunities as well as similar flight distances. Furthermore, in addition to the degree of deviation between the models/data sets, the direction of the deviation should also be considered in more detail in further investigations.

## Supporting information

S1 Table Breakdown of the biotope types. (XLSX)

S2 Table Allocation of biotope types to groups. (XLSX)

S3 Table Allocation of CLC classes to groups. (XLSX)

S4 Table List of wild bee species. (XLSX)

S5 Table Land use classes (biotop class dataset) with associated nesting suitability and floral resources for wild bees. (XLSX)

S6 Table Land use classes (CLC Class Dataset) with associated nesting suitability and floral resources for wild bees [18]. (XLSX)
